# The Emergence of Pan-Cancer CIMP and Its Elusive Interpretation

**DOI:** 10.3390/biom6040045

**Published:** 2016-11-22

**Authors:** Brendan F. Miller, Francisco Sánchez-Vega, Laura Elnitski

**Affiliations:** 1 National Human Genome Research Institute, Rockville, MD 20852, USA; millerbf2@mail.nih.gov; 2 Memorial Sloan Kettering Cancer Center, New York, NY 10065, USA; sanchezf@cbio.mskcc.org

**Keywords:** pan-cancer, CpG island methylator phenotype, DNA methylation

## Abstract

Epigenetic dysregulation is recognized as a hallmark of cancer. In the last 16 years, a CpG island methylator phenotype (CIMP) has been documented in tumors originating from different tissues. However, a looming question in the field is whether or not CIMP is a pan-cancer phenomenon or a tissue-specific event. Here, we give a synopsis of the history of CIMP and describe the pattern of DNA methylation that defines the CIMP phenotype in different cancer types. We highlight new conceptual approaches of classifying tumors based on CIMP in a cancer type-agnostic way that reveal the presence of distinct CIMP tumors in a multitude of The Cancer Genome Atlas (TCGA) datasets, suggesting that this phenotype may transcend tissue-type specificity. Lastly, we show evidence supporting the clinical relevance of CIMP-positive tumors and suggest that a common CIMP etiology may define new mechanistic targets in cancer treatment.

## 1. Introduction

Through the auspices of large-scale consortium projects, such as The Cancer Genome Atlas (TCGA), the International Cancer Genome Consortium, Cancer Epigenome, and the International Human Epigenome Consortium, our understanding of cancer processes and epigenetic landscapes has vastly accelerated in the past five years. Given that cancer develops through defects in the controls that regulate cellular processes such as proliferation, cell death, and metastasis [1,2], the spectrum of events that causes accumulation of genetic mutations and changes the expression or function of genes and their downstream protein products has significantly widened in consideration [3]. We now know that the collection of molecular disruptions that contribute to oncogenesis includes not only mutations and copy number alterations but also changes to the cellular epigenetic state [4]. Indeed, a large proportion of mutations in almost all cancer types affect epigenetic regulators, implying that epigenetic dysregulation is inherently linked to oncogenesis [2,5].

By defining epigenetic patterns in tumors, we may be able to improve both the detection and treatment of cancer [6,7,8]. Some DNA methylation signatures are stable, easily detectable, and present across subgroups of tumors [9,10], suggesting that informative loci can be used to distinguish between cancerous and non-cancerous cells [11]. Because epigenetic dysregulation can affect gene transcription, identifying affected loci gives clues to tumorigenic processes. This information may ultimately inform the use of drugs that target epigenetic pathways, which are increasingly being viewed as powerful adjuncts to combination drug therapies [12,13]. Thus, a better understanding of cancer epigenetic dysregulation can be leveraged to identify tumor subtypes, improve precision medicine regimens, and lay the groundwork for diagnostic and screening purposes.

In this review, we focus on the CpG island methylator phenotype (CIMP): a pattern of extensive DNA hypermethylation at cytosines located in CpG islands (CGIs). The CIMP subtype has been documented in some cancers including colorectal, glioma, leukemia, and breast [14]. However, whether CIMP represents a distinct subtype in cancers from all tissues has been a controversial subject, plagued by inconsistencies in classification schemes and a lack of clear-cut guidelines to define the phenomenon. The availability of large (methylation and other) datasets courtesy of the aforementioned cancer consortia has allowed the field to conduct statistically powerful analyses aimed at identifying CIMP. Now, not only can we clearly demarcate CIMP subtypes in multiple cancer types, but we can also begin to explore the molecular underpinnings that drive the phenotype. Here, we present evidence that establishes CIMP as a pan-cancer phenomenon rather than a tissue-specific mechanism and explore the relevance of driver mutations to this phenotype. Our goal is to summarize the state of the field and highlight future directions in this rapidly evolving area of study.

## 2. Identification of CIMP in Colorectal Cancer

CIMP was first characterized in 1999 by Minoru Toyota et al. in primary colorectal cancer (CRC) samples [15]. At the time, abnormal methylation at CGIs had been proposed as an alternative mechanism of gene inactivation in neoplasia [16], and the authors sought to characterize these patterns on a genome-wide scale. Using a PCR-based method they identified 26 CGI sequences that were methylated in primary CRC tumors (known as MINTs). Of these, seven were specifically methylated in CRCs in an age-independent manner. When applied to a larger panel of 50 primary CRCs, these seven MINTs defined two groups of samples: those with three or more methylated sites or those without methylation at any of the seven MINTs. Most of the samples in the former group displayed methylation at the tumor suppressor genes *p16* and thrombospondin 1 (*THBS1*), and the mismatch repair gene MutL homolog 1 (*MLH1*). This led to the hypothesis that a “methylator phenotype” is an alternative driver of tumorigenesis; in this model, hypermethylation at CGIs silences tumor suppressor genes, which causes the accruement of mutations that inevitably lead to cancer.

The existence of a distinct CIMP subtype in CRC remained controversial for many years due to the arbitrary definition(s) used to define it. A study by Yamashita et al. [17] challenged the existence of CIMP by reporting a gradual accumulation of methylation in CRC samples. The authors could not identify a clear boundary defining CIMP-positive and CIMP-negative tumors and concluded that age explained the increase in methylation. However, later reexamination of the data indicated that tumors clustered with respect to methylation of specific genes, suggesting that CIMP could be discerned using appropriate statistical analyses [18]. Additional support came from Weisenberger et al. [19], who performed unsupervised hierarchical clustering on 295 primary CRC samples using the methylation status at 195 CGIs. Filtering CGIs to remove methylation events associated with aging revealed a distinct CIMP-positive cluster of samples, which shared proto-oncogene B-Raf (*BRAF*) V600E mutations and microsatellite instability (MSI). Interestingly, the researchers identified a secondary cluster of samples that displayed an overall intermediate level of methylation across the selected CGIs; this methylation pattern was associated with V-Ki-ras2 Kirsten rat sarcoma viral oncogene homolog (*KRAS*) mutations. Others have subsequently defined CRC subtypes including CIMP-high, CIMP-low and CIMP-zero [20,21,22,23], in which this KRAS-positive group with attenuated CIMP is labeled CIMP-low. Nevertheless, Weisenberger et al. initially noted that inclusion of this group gave the appearance of a continuous gradient in the methylation signal. We note that the interpretation of the patterns, whether dichotomous or continuous, is relevant for formulating precise mechanistic hypotheses.

**Figure 1:**
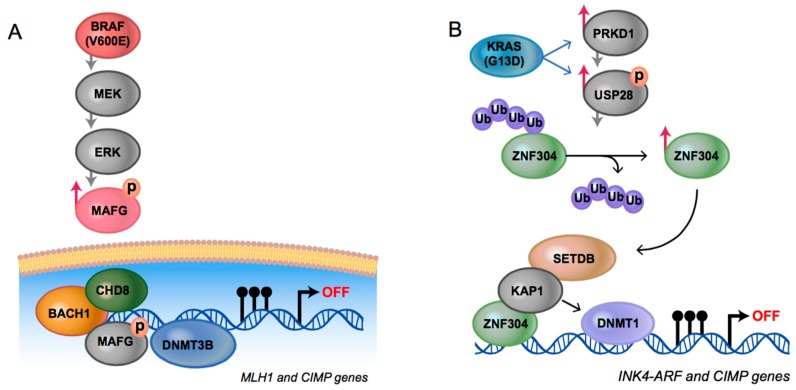
Mechanisms establishing CpG island methylator phenotype (CIMP) in colorectal cancer: (**A**) Model reproduced from [24] with permission from Elsevier; protein kinase B-Raf (BRAF) V600E increases MAPK/ERK kinase (MEK)/extracellular signal-regulated kinase (ERK) signaling and causes levels of MAF BZIP transcription factor G (MAFG) to increase via phosphorylation, which protects it from polyubiquitination and subsequent degradation via the proteasome. MAFG directs a transcriptional repression complex that includes the DNA methyl transferase DNMT3B to CIMP-associated genomic loci; (**B**) Model reproduced from [25] published by eLife Sciences Publications Ltd.; activated V-Ki-ras2 Kirsten rat sarcoma viral oncogene homolog (KRAS) increases levels of zinc finger protein 304 (ZNF304) through transcriptional upregulation of the deubiquitinase ubiquitin specific peptidase 28 (USP28) and the serine/threonine kinase protein kinase D1 (PRKD1). ZNF304 recruits a transcriptional repression complex that includes the DNA methyl transferase DNMT1 to CIMP associated genomic loci. Illustrations reproduced by Darryl Leja. BACH1: BTB domain and CNC homolog 1; CHD8: chromodomain-helicase-DNA-binding protein 8; INK4-ARF: inhibitors of CDK4-ADP ribosylation factors; KAP1: KRAB-associated protein-1; MLH1: MutL homolog 1; SETDB: SET domain bifurcated 1; Ub: ubiquitin.

The well-supported existence of CIMP in CRC enabled better characterization of the molecular underpinnings driving the *BRAF* CIMP-high and *KRAS* CIMP-low CRC subtypes (Figure 1). Regarding the former, it was shown that the oncoprotein BRAF V600E phosphorylates the transcriptional repressor MAF BZIP transcription factor G (MAFG), leading to the assembly of a repressor complex that directs the DNA methyl transferase, DNMT3B, to methylate specific promoter CGIs [24]. In the latter case, it was demonstrated that the overactive KRAS protein transcriptionally upregulates protein kinase D1 (*PRKD1*) and the deubiquitinase ubiquitin specific peptidase 28 (*USP28*), which prevents degradation of zinc finger protein 304 (ZNF304) [25]. As a transcriptional repressor, ZNF304 recruits DNMT1 to select target sites, leading to promoter methylation and transcriptional silencing. Interestingly, the two pathways are not interchangeable; knockdown of *MAFG* in the *KRAS* mutant cells or *ZNF304* in the BRAF V600E positive cells did not inhibit the promoter methylation events [24]. Moreover, there was no physical association seen between the MAFG methyltransferase recruitment complex and target genomic sites in the *KRAS* mutant cells. Conversely, there was no physical association between the ZNF304 complex and target sites in the BRAF V600E cells. Taken together, the authors concluded that disparate oncogenic pathways converged on shared genomic target sites. However, the regulatory process that guided the *trans*-acting factors and associated complexes to these loci remains unknown.

## 3. Evidence for Pan-Cancer CIMP

Since its discovery in CRC over 16 years ago, the concept of CIMP has undergone constant evolution, leading to anecdotal reports in multiple cancer types, with little or no overarching consistency. For example, CIMP has been described in bladder [26], breast [27,28,29], endometrial [30,31], gastric [32,33,34,35,36,37], liver [38,39,40,41], lung [42,43], ovarian [44,45], pancreatic [46], prostate [47], and kidney cancer [48], as well as gliomas [49,50,51], leukemia [52,53,54,55], melanoma [56], duodenal adenocarcinomas [57], adrenocortical carcinoma [58], and neuroblastoma [59,60]. Many of these studies rely on the minimalist gene panels described by Toyota and Weisenberger, with inconsistent use of thresholds to assign tumors as CIMP positive. These panels were designed specifically for defining CIMP in CRC using methods that were limited in the genomic space queried and may not be applicable towards other cancers. When examined carefully, different tumor types with CIMP designations do not carry a majority of overlapping methylated loci, making it difficult to decide whether CIMP is a pan-cancer event caused by a universally shared mechanism or is an individualistic occurrence observed in each tumor type [14]. Reconciliation of these options will require the research community to agree on a default set of criteria to classify CIMP that is independent of cancer type.

Illumina Infinium HumanMethylation27k and HumanMethylation450K array data, available through consortia such as TCGA [61], have enabled researchers to apply an unbiased and unsupervised hierarchical clustering approach to CIMP classification of large numbers of samples, both in individual and multiple cancer types. Noushmehr et al. [49] performed unsupervised hierarchical clustering on methylation profiles of 272 glioma tumors and revealed a CIMP subgroup that was enriched with isocitrate dehydrogenase 1 (*IDH1*) mutations. Fang et al. [28] identified strong evidence for CIMP in breast cancer after performing hierarchical clustering on 39 primary tumors, and validated these results on an independent group of 132 samples using only three loci found to be hypermethylated in their initial analysis. Kolbe et al. [45] reported a methylator phenotype using unsupervised hierarchical clustering on 168 ovarian and uterine endometrial tumors. The endometrioid endometrial samples displayed widespread hypermethylation in CGIs, regardless of whether they were metastatic endometrioid tumors that originated in the uterus and migrated to the ovary, or primary tumors in either tissue. Thus, the epigenetic landscape in these tumors represented a disruption in a DNA methylation pathway and not a tissue type-specific signature. Importantly, several studies show that CIMP correlates with clinicopathologic features [14] and is therefore clinically relevant. In ovarian and uterine cancers, a historical distinction of low-grade type I and high-grade type II tumors corresponded to lower and higher levels of tumor aggressiveness, endometrioid and serous subtypes, and presence and absence of CIMP, respectively [45].

**Figure 2:**
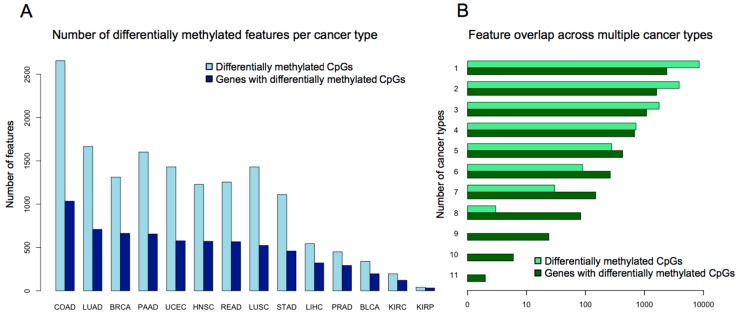
Number of differentially methylated genomic loci across cancers: (**A**) Figure generated using data from [62]. The vertical axis represents total count. For each cancer, the differentially methylated CpGs were defined as those with average methylation levels above 25% in tumors and less than 5% in controls; (**B**) Figure generated using data from [62]. The horizontal axis represents the number of features (either differentially methylated CpGs or genes that contain differentially methylated CpGs) that are shared by a number of cancer types that is greater than or equal to the value shown in the vertical axis. COAD: colon adenocarcinoma; LUAD: lung adenocarcinoma; BRCA: breast invasive carcinoma; PAAD: pancreatic adenocarcinoma; UCEC: uterine corpus endometrial carcinoma; HNSC: head and neck squamous cell carcinoma; READ: rectum adenocarcinoma; LUSC: lung squamous cell carcinoma; STAD: stomach adenocarcinoma; LIHC: liver hepatocellular carcinoma; PRAD: prostate adenocarcinoma; BLCA: bladder urothelial carcinoma; KIRC: kidney renal clear cell carcinoma; KIRP: kidney renal papillary cell carcinoma.

**Figure 3:**
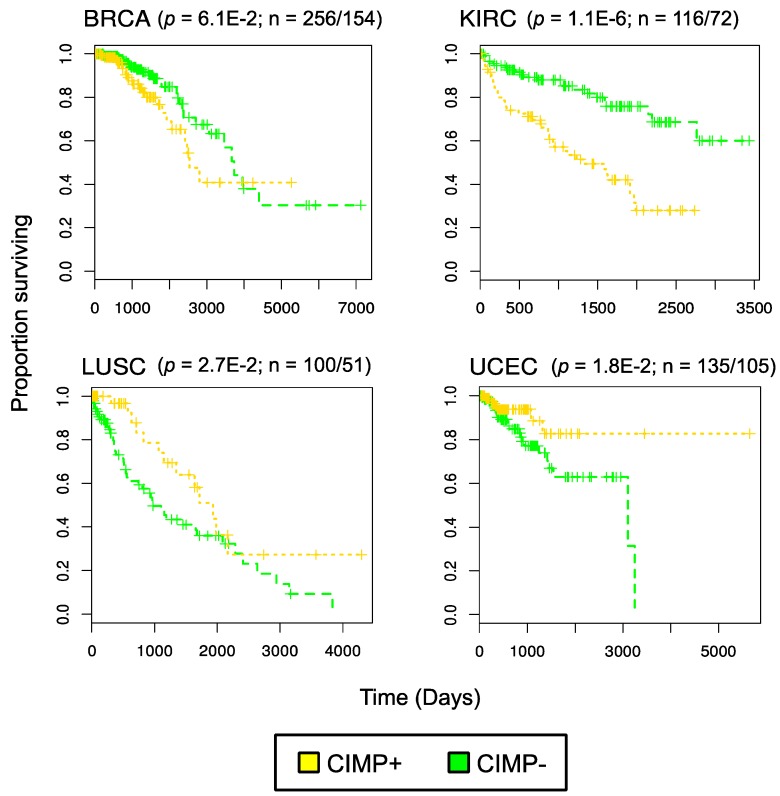
Survival curves in four cancer types based on CIMP: figure reproduced from [62] and published by BioMed Central. Data generated using The Cancer Genome Atlas clinical data for these cancer types. *p* values were determined using a log-rank test for survival differences. Ratio (n) indicates number of CIMP-/CIMP + samples.

The above examples gave a clear opportunity to address commonalities in CIMP methylation targets as defined across multiple individual studies. A formalized pan-cancer analysis came from our group, who analyzed genome wide patterns of CGI hypermethylation in 5253 solid tumor samples belonging to 15 TCGA cancer types [62]. Using Illumina HumanMethylation450K data, we used the same methodology across all cancer types to select a set of distinctive high variance probes. Specifically, these probes displayed minimal methylation in controls and high methylation in tumors. The number of CpG probes differentially methylated between tumors and corresponding normal samples and the associated genes overlapping these probes for each cancer is illustrated in Figure 2A and suggests that a large number of genomic loci are targeted for methylation. For each cancer type, the high variance probes were used to stratify samples into three classes with *k*-means clustering, using the mean methylation values across the differential probes. This approach defined CIMP-positive, CIMP-intermediate, and CIMP-negative subgroups for 14 of the 15 cancer types investigated; thyroid cancer alone had no differentially methylated probes. This uniform appraisal of all TCGA cancer types showed that high average levels of methylation across the tumor target sites compared with normal samples defined CIMP. We went on to define a minimal, semi-overlapping set of 89 pan-cancer methylation sites that could be used to classify tumors as CIMP-positive or negative in the majority of cancers queried. Importantly, our CIMP labels obtained on TCGA CRC samples agreed well with the CIMP labels assigned by Weisenberger to CRC tumors (as shown in [62]). Consistent with the above observations, Moarii et al. [63] defined pan-cancer CIMP in five cancer types using 2090 TCGA tumors. The authors classified samples as either CIMP-positive or CIMP-negative via unsupervised hierarchical clustering using the top 5% of variably methylated CGIs for a given cancer type. By comparing the sites implicated in CIMP-positive tumors for each cancer type, the authors assembled a common set of 89 hypermethylated CGIs associated with 51 genes. Paired with our lab’s findings, these results corroborate the idea that a collection of loci is consistently aberrantly methylated in a subset of tumors across many tissues (Figure 2B). Thus, TCGA datasets enable the research community to formulate hypotheses about common mechanisms of CIMP across cancer types. Furthermore, a refined ability to detect CIMP may be useful for stratifying tumors in clinical trials, enabling researchers to investigate CIMP-specific responses. Notably, our studies documented a gradient of increasing DNA methylation across CIMP-positive and CIMP-intermediate samples for all the sets of differentially methylated probes, similar to observations by Weisenberger in CRC [19]. However, we were able to show overall survival curves exhibiting significant differences based on CIMP-positive and CIMP-negative subgroups for four cancer types (breast invasive carcinoma, BRCA; kidney renal clear cell carcinoma, KIRC; lung squamous cell carcinoma, LUSC; uterine corpus endometrial carcinoma, UCEC) (Figure 3); overall survival curves for luminal A and luminal B subtypes in BRCA also differed by CIMP.

In summary, technologies to assay methylation across the entire genome have allowed researchers to employ data-driven and tissue type-agnostic approaches to describe CIMP. Unsupervised hierarchical clustering has revealed CIMP in multiple cancers and demonstrated that a common set of CpGs is targeted. Thus, we now have a better method to identify CIMP, one that is free from the biases of gene panels. These patterns of hypermethylation are strong predictors of prognosis (at least for a handful of cancers), independent of patient age or tumor stage, supporting the idea that CIMP represents a specific molecular subtype. Now that groups of CIMP tumors can be confidently identified, other datasets need to be leveraged to elucidate the molecular commonalities underlying the etiology of this subtype, as well as the clinical ramifications.

## 4. Shared Pathways and Common Mechanisms for CIMP

The reproducible nature of the hypermethylation patterns in CIMP tumors within and across tissues indicates that this is not a stochastic process. However, uncovering the mechanism responsible has proved challenging. For example, researchers have not been able to identify a universal, causal CIMP driver mutation set. Although shared mutations can be observed in CIMP tumors that originate from different tissues, as we found in Sánchez-Vega et al. [62], not all tumors that harbor a given mutation display CIMP. Furthermore, no functional relationship to CIMP has been identified for many of the driver mutations. Thus, these associations are regarded as mere correlations at present. Assuming that CIMP is established through a pan-cancer process, the absence of evidence for causal mutations suggests that CIMP most likely occurs through functions shared along a molecular pathway, rather than through mutations in a single driver gene.

If separate pathways can lead to CIMP, they must converge to achieve consistent patterns of methylation. Where this convergence occurs remains unclear, but connections between pathways previously thought to be unrelated are emerging. For example, increased KRAS signaling through the extracellular signal-regulated kinase (ERK) pathway inhibits expression of the DNA demethylase ten–eleven translocation enzyme 1 (TET1) and promotes tumor formation [64]. Glioma CIMP tumors are strongly associated with an *IDH1* gain of function mutation, resulting in production of an oncometabolite that inhibits DNA demethylation enzymes (TETs) [65]. This may hint at a link between CIMP CRC tumors, which are typically associated with *BRAF* and *KRAS* mutations but not *IDH1* or *TET* mutations, and CIMP gliomas, which are associated with *IDH1* mutations [50] but not *BRAF* or *KRAS* mutations. However, the question remains whether the mechanism establishing CIMP across multiple tissue types represents a pan-cancer phenomenon or a tissue-specific one.

Pan-cancer CIMP reflects a common trend in cancer genomics: on the one hand, tumors from the same tissue of origin can display strikingly different genomic alterations, but, on the other hand, similar patterns are shared between tumors from different tissues [66,67]. Consistent with this idea, Ciriello et al. [68] characterized ≈500 functional alterations (recurrent somatic mutations, copy number alterations, and gene silencing DNA methylation events) in 3299 tumors comprising 12 cancer types and observed a strong inverse relationship. Tumors with high levels of genomic instability were enriched for either somatic mutations (i.e., “mutator class”) or copy number alterations (i.e., “copy number class”) but not both. This trend was also independent of tissue of origin. The mutator class included many gliomas, CRCs, and uterine carcinomas, as well as acute myeloid leukemia—namely, cancer types in which CIMP has been previously identified. Deeper analysis of the mutator class revealed two subclasses—one defined by altered phosphoinositide 3-kinase (PI3K)– protein kinase B (Akt) signaling and highly enriched for MSI/BRAF-mutated CRC samples, the other defined by KRAS mutations, altered Ras and Wnt signaling, and adenomatous polyposis coli (*APC*) mutations. Interestingly, uterine and ovarian serous samples, which have been classified as primarily CIMP-negative [45], were assigned to the copy number class, whereas endometrioid tumors fell into the mutator class. After integrating our pan-cancer CIMP labels with the selected functional events identified by Ciriello and generating recursive binary decision trees, we also found that CIMP-positive and CIMP-negative samples were enriched in the mutator and copy number class, respectively [62]. Collectively, these results indicate that some CIMP tumors are characterized by MSI and excessive somatic mutations, but not copy number alterations, and that a few dysregulated pathways are common across cancer types. Whether CIMP leads to an increase in somatic mutations is unclear, but because DNA methylation changes are early events in tumorigenesis [69] and some CIMP-targeted genes include members of the mismatch repair machinery (*MLH1*, for example), this remains a possibility.

Several functional connections suggest that the cell’s metabolic state may play a role in establishing CIMP, including the observation that mitochondrial-acting *IDH1* mutations are sufficient to establish CIMP in glioma [50], they are mutually exclusive with *TET* mutations, and both are tightly associated with CIMP in at least two different cancers [70,71] including glioma and adult myeloid leukemia. In this capacity, metabolic enzymes in the cell tightly control the flux of substrates utilized by epigenetic enzymes to add and remove chemical modifications [72,73]. Under normal conditions, IDH enzymes reversibly convert isocitrate, an intermediate in the citric acid cycle, to α-ketoglutarate (2KG), which can be used by TET enzymes to convert methyl cytosines to hydroxymethyl cytosines [74]. These bulky groups are subsequently replaced with unmethylated cytosines as part of the active DNA demethylation pathway [75]. Thus, both IDH and TET are indispensable in the process of DNA demethylation, and, when DNA demethylation cannot proceed normally, oncogenesis may result [76]. The most commonly reported *IDH* mutation, especially in some CIMP tumors, is R132H [65]. This mutant form of the protein allows production of the oncometabolite 2-hydroxyglutarate (2HG), which inhibits TET enzymes and thus increases DNA methylation. Another piece of evidence implicating the cell’s metabolic state in CIMP comes from mutations in succinate dehydrogenase, another mitochondrion-associated enzyme. A high ratio of succinate to fumarate, two additional downstream intermediates in the citric acid cycle, can inhibit 2KG dependent enzymes (like TETs). Thus, alterations in mitochondrial processes (and the metabolic state in general) may be a common trend in CIMP. Mutations in mitochondria, which are well documented in cancer, can also result in fluctuations of intracellular Ca^2+^ [77]. Intriguingly, DNMTs, which typically attach methyl groups to cytosines, have been shown to remove the groups in vitro when the concentration of Ca^2+^ is high [78]. Thus, altered Ca^2+^ concentrations could result in aberrant patterns of methylation. Given that cancer cells are typified by altered metabolism [72], the above observations encourage a comprehensive comparison between the metabolic profiles of CIMP positive tumors and CIMP negative ones.

In conclusion, we take the position that CIMP represents a disruption to epigenetic regulation as a whole (via DNA mutations, altered metabolism, etc.), which establishes aberrant patterns of methylation that may modulate the biology of the tumor. In this light, CIMP is analogous to other genetic phenotypes such as MSI, in which a disruption to a pathway causes widespread genome instability that can contribute to tumorigenesis. Whether this epigenetic disruption occurs at the individual gene level (as seen in the case of CIMP associated mutations to *IDH* and *TET* genes) or at a higher pathway level remains to be seen and warrants further exploration.

## 5. Passenger and Driver Events in DNA Methylation

The question remains: Does CIMP contribute to carcinogenesis or is it merely a surrogate of epigenetic dysregulation? Though reversible, epigenetic events can persist through multiple cell divisions and they represent one route toward the formation of cancer [2]. Hypermethylation of cell-cycle regulator genes such as *Rb*, *APC*, *p15/CDKN2B*, and *p16/CDKN2A*, and DNA repair genes such as *BRCA1* and *MLH1*, have been reported in various cancers [76]. However, in the case of frequent and widespread methylation events in CIMP tumors, very few clearly established “driver” hypermethylation events of tumorigenesis (which we define as epigenetic silencing of active genes leading to cancer initiation and progression) are documented—the most common being hypermethylation of *MLH1* in CRCs, which rapidly increases the rate of malignancy [79]. However, the role of *MLH1* in the causality of CIMP has been questioned [80]. Perhaps the processes establishing tumorigenesis via widespread CpG island methylation have more complex interpretations than directly modulating the expression of downstream genes and may rarely silence tumor suppressor genes.

A model in which a handful of “driver” methylation events exists against a background of many “passenger” methylation events echoes that proposed for genetic mutations in tumors [81]. Supporting the high prevalence of passenger methylation events, Sproul et al. [82] analyzed 1154 tumor samples from seven different tissues and identified 1009 genes that had hypermethylated CpG promoters in at least one type of cancer. Of these genes, 220 were consistently methylated in multiple cancer types, but most were variably methylated between cancers, and roughly half were methylated in only one type. Genes with consistently methylated promoters in multiple cancers showed expression patterns that tended to be tissue-specific, whereas variably methylated genes were expressed in more tissues. The researchers concluded that genes targeted for hypermethylation in cancer were already repressed in pre-cancerous tissue. This is consistent with the observation that sets of hypermethylated genes are also enriched for polycomb repressive complex binding and the presence of bivalent histone modifications [83,84], which are associated with lineage-specific and differentially activated or repressed genes. Genes that are targeted for hypermethylation in cancer and are expressed at low levels in normal tissue have been noted in several other reports [62,85]. Thus, with respect to gene promoters, most methylation events appear to be passenger events (with no apparent contribution towards tumorigenesis) potentially mediated by polycomb repressive complex. Nonetheless, epigenetic silencing of tumor suppressor genes does occur [76], and it remains to be seen whether this targeting is a function of polycomb repressive complex dysregulation.

Despite extensive evidence pointing to a repressive role for DNA methylation, not all promoter methylation events cause gene silencing [86], so methylation events in CIMP that “drive” oncogenesis may exert their effect by other means (through reorganization of enhancer-promoter interactions, for example). Methylation of CpGs in CTCF binding motifs can disrupt genome topology and lead to activation of oncogenes, at least in *IDH*-mutant gliomas [87]. This is consistent with a role for chromatin topology in the regulation of genomic imprinting [88] and its role in the formation of large hypomethylated regions in CRCs that correspond to nuclear lamina-associated domains [89]. Lamina-associated domains are regions of chromatin located near the periphery of the nucleus and tethered to the intermediate filament lamin. In cancer, these chromatin domains can become disorganized and may also become associated with large blocks of organized chromatin lysine modifications [4,90]. These observations should inspire an in-depth comparison of differences in chromatin topology between CIMP-positive and CIMP-negative tumors within and across cancer types.

## 6. Conclusions

The story of CIMP illustrates the utility of using unbiased genome-wide approaches to characterize distinct groups of samples. Initially, researchers based their classification schemes on limited panels of hypermethylated genes biased towards CRC. With the advent of genome-wide methylation profiling technologies, tissue type-neutral approaches were undertaken to refine gene panels, improve confidence in CIMP assignment, and allow characterization of the CIMP tumor subtype at the molecular level. Nevertheless, the interpretation of pan-cancer CIMP and the mechanism(s) establishing it remains preliminary and perhaps controversial. Although the relationship between driver mutations and CIMP is correlational at this time, some driver mutations have been shown to have a plausible route to changes in DNA methylation. However, how multiple driver mutations converge into pathways that affect DNA methylation is not obvious. Nonetheless, the same mutations are found in distinct cancer types that affect Wnt signaling or PI3K pathways and associate with CIMP, and in other cases metabolic processes produce inhibitors of DNA and histone demethylases. Given the differential clinical subtypes noted in CRC, as well as uterine and ovarian cancers, and their distinctive CIMP subtypes, it is plausible that other tumor types with reported CIMP patterns can be subdivided into distinct clinical subtypes as well. These distinctions may be important to reduce the heterogeneity seen in collections of tumor samples, thereby creating more homogeneity in responses to therapeutic approaches in clinical trials. Moving forward, steps should be taken to define distinct classes of tumors in terms of their molecular dysregulation and to design effective probes to query molecular dysfunction. DNA methylation is an appropriate candidate for these purposes because it not only represents a measurable readout of the genomic landscape but also can contribute to cancer progression. Additionally, it can be used as a biomarker in blood-based detection assays, allowing for minimally invasive and early-stage cancer screening [91].

In conclusion, we hope that these observations will reinvigorate interest not only in interpreting and eventually treating CIMP but also in understanding cancer epigenetic dysregulation in general.
